# Expression of Concern: Associations of *MMP-2* and *MMP-9* gene polymorphism with ulinastatin efficacy in patients with severe acute pancreatitis

**DOI:** 10.1042/BSR20160612_EOC

**Published:** 2025-10-09

**Authors:** Guo-Dong Zhen, Lian-Bin Zhao, Shan-Shan Wu, Ming-Yu Chen, Zhen-He Li, Sheng-Zhi Zhou, Zhen-Fu Li

**Affiliations:** Department of Emergency, Yishui Central Hospital of Linyi, Linyi 276400, Shandong Province, China

*Bioscience Reports* has been made aware of potential issues surrounding the scientific validity of this paper and is hence issuing an Expression of Concern to notify readers whilst the Editorial Office investigates. Irregularities have been noted in the Western Blots of Figure 1B and C. Specifically, there are background details in Figure 1B that unexpectedly repeat, and a region of the CC column of Figure 1C that has a smudged appearance. The authors have been contacted for comment, and we have not yet received a response or the requested raw data.

